# Book Reviews

**Published:** 1903-07

**Authors:** 


### BOOK REVIEWS.

The Internal Secretions and the Principles of Medicine. By Charles E. de M. Sajous, M. D., Fellow of the College of Physicians of Philadelphia; formerly Lecturer on Laryngology in Jefferson Medical College, and Professor of Laryngology and Dean of the Faculty in the Medico-Chirurgical College. Volume First. Octavo, pp. 826. With 42 illustrations. Philadelphia: F. A. Davis Co. 1903.

This work will attract attention from the somewhat startling nature of its claims, yet gradually many of its assertions have already been admitted as among the tenable truths of science. The physiology of the adrenals, with which the first chapter is concerned, is viewed from the standpoint of clinical pathology. To determine the relative functional importance of the suprarenal glands in man and the lower animals is an essential point to settle, yet it is a field of study, beset with obstacles. Sajous has made this question one of great study, but thus far has not reached absolute definiteness as to all the points involved. He, however, affirms that "the physiological functions of the adrenals are sufficiently similar in all vertebrates to warrant the use of experimental data obtained with lower animals in the study of these organs in man." There are many points of great interest connected with the study of the adrenals that we must pass without even commenting upon.

The thyroid and thymus glands and the adrenals considered in their relationship, presents another question of interest, and one that furnishes opportunity for much speculation. It has been the accepted opinion for some time that myxedema and cretinism are due to insufficiency of secretion of the thyroid gland, and Sajous concludes that these two diseases are due to insufficiency of the adrenals, primarily, which causes diminution or absence of secretion in the thyroid.

After discussing the relations of the anterior pituitary body, the thyroid gland, and the adrenals, as parts of an autonimous system, the author takes us to the pathogenesis of acromegaly, of which he distinguishes three forms,—one due to overnutrition of predisposed pituitary through continuous overactivity of the adrenals and maintained by chronic toxemia; a second, due to persistence of the thymus, brought on by overstimulation of the adrenals; and a third, due to morbid processes, such as tumors and the like, in the pituitary itself. On page 234, a singular error has crept into the text by omission of the words "the temperature" (of a dog, etc.), and again a little farther on, "when the muscles contraction as follows," is an imperfect sentence that indicates another omission. These, of course, will be corrected in the next edition.

The oxidising substance is discussed in much detail and is considered very fully as subserving the physiological needs of the functions of the stomach, liver, heart, lungs, intestinal tract; in short all the organs concerned in air or food nutrition. Perhaps, if the part played by the adrenals in the nourishment and waste—in the metabolism—of the body, as conceived by the author, becomes established, there will be more precision in the therapeutics of nutrition, indigestion, and the wasting diseases. Sajous becomes very interesting—intensely so, we may say—when he presents the relation the adrenal secretion bears to the heart, and discourses upon the sources of the heart's energy. This is a field too little understood because too little explored. These are strenuous days and the heart feels the strain intensely, often giving out before it should or would if its energy were properly conserved. Carrying the subject forward through the nerve centers, the author offers many thoughts of interest because of their important bearing on the preservation of health and longevity. Their very novelty will attract and if their practical establishment shall finally take place, a valuable addition to the science of medicine will have been made.

The means offered by nature to protect herself are many, but of them all the immunising processes through which the body is protected against infection are, perhaps, the most remarkable. Sajous takes this up in a discussion of the relations of the adrenal system to antitoxic serum, and explains how poisons are converted into harmless substances through the agency of the adrenals. In the course of this investigation the leukocyte in its relations to life and organic functions plays an important part. Just now the subject of leukocytosis is being discussed in almost every medical and surgical phase, and it is quite germane to the question that Sajous has written in this treatise so much of value. That the leukocyte is an important factor in immunity is more than probable.

It would be interesting to pursue this entire field in detail, but we are reminded of the fact that a review must be compact, not diffuse, in order to command even moderate attention. The functions of the ductless glands as oxygen producing or supplying agents is important and, if completely and wholly true, makes the more important organs take secondary place, to work out their destiny at the behest and under the mandatory influence of these hitherto little understood almost insignificant bodies.

The entire volume betrays a patient study and a tireless energy that are pleasant to contemplate. It is withal, a fascinating book that will assuredly attract attention in the laboratory and in the clinic. It invites the investigator of cause to prove or disprove its statements, its inferences, or its asserted facts. It beckons onward into a mazy labyrinth of unexplored or but partially explored physiologic and pathologic jungles, where a reward awaits that shall amply repay the student for his work. The artistic illustrations deserve, in conclusion, a word of praise. They are much above the average in delineating the text and in perfection of color, tint, and execution.

---

HARRINGTON'S HYGIENE. A Manual of Practical Hygiene for Students, Physicians and Health Officers. By CHARLES HARRINGTON, M. D., Assistant Professor of Hygiene in the Medical School of Harvard University. New (2d) edition, revised and enlarged. In one octavo volume of 755 pages, illustrated with 113 engravings and 12 full-page plates in colors and monochrome. Philadelphia and New York. Lea Brothers & Co. 1903. [Cloth, $4.25 net.]

The prime object of the author of this work was to create a textbook for students and it was our privilege as well as pleasure more than a year ago (November, 1901, p. 312), upon or soon after the first edition appeared, to invite attention to the success which he had attained in this respect. In the interval there has been much progress in the field of hygiene, but Harrington has kept pace with it, and in this new edition has recorded many facts not contained in the first.

The changes in this issue have required additions to the extent of seventy pages, and a substraction or elimination of some thirty pages, leaving a net gain of about forty pages, though there is no apparent increase in the size of the book. While many chapters have been in part rewritten, the chief addition results from a new chapter dealing with the relation of insects to human diseases. This subject though not entirely new has received only recently that scrutiny which its importance requires. It was not until bacteriology threw light on the methods of infection carriers, that the rôle played by insects came even to be suspected. Harrington's chapter deals so intelligently with this whole question that we had almost pronounced it the best exposition of it, in condensed form, that has yet appeared. The eight or nine illustrations in this chapter are helpful in a marked degree to the study of the mosquito, especially to a differentiation of the species.

The pages eliminated related chiefly to quarantine law and some other obsolete material, but many other changes permeate the treatise, making it quite abreast of the immediate present in all particulars.

We observe, on page 597, that credit is given "Dr. P. C. Harris, U.S.A.," for a plan of a sanitary camp. Captain P. C. Harris, Q.M., U.S. Army, then stationed at Fort Porter, prepared a plan of such a camp and demonstrated it at a meeting of the Buffalo Sanatory Club, December 14, 1898. This was published in the BUFFALO MEDICAL JOURNAL, February, 1899, which is the proper reference to Capt. Harris's drawing, one that is quite original and the most complete of its kind.

All through Harrington's treatise the genius of hygiene pervades, and it gives equal scientific thought whether dealing with foods, school sanitation, or personal hygiene; whether handling the most complex problems of public sanitary science, or those relating to the personal health of the individual. For these and other reasons it may be regarded as quite one of the best expositions of hygiene in its broadest application, that has been published.

Surgical Anatomy. A Treatise on Human Anatomy in its Application to the Practice of Medicine and Surgery. By John B. Deaver, M. D., Surgeon-in-Chief to the German Hospital, Philadelphia. In three volumes. Illustrated by 499 plates, nearly all drawn for this work from original dissections. Volume III. Abdomen; pelvic cavity; lymphatics of the abdomen and pelvis; thorax; lower extremity. Philadelphia: P. Blakiston's Son & Co. 1903. (Price: cloth, $21.00; full sheep, $24.00; half Russia, gilt, marbled edges, $27.00 net.)

The American medical profession is under great obligations to Professor Deaver for the most comprehensive and best surgical anatomy that has been published as yet,—indeed, we might include the profession of medicine throughout the civilised world in this statement. At all events, we are quite sure that all scientific physicians, teachers, and students everywhere will unite in thanking Dr. Deaver for his painstaking and valuable work.

The author offers an apology for the delay of this volume, and proceeds to give some of the reasons that caused it, but he need not feel disturbed over the length of time that has been required to prepare the book. It is an old maxim that "a work done well is done soon enough," and in this instance it seems to have special application. The only wonder is that such a work, involving such an outlay of time, such painstaking dissections, and such minutæ in engraving, could be completed even so soon. The viscera of the cavities are so difficult to obtain in suitable forms for the artist that, of necessity, many cadavers must be examined, not a few of which had to be discarded.

In spite of all the difficulties in the way, the work is now complete in its three volumes, and is a momument not only to the author but to American anatomical science that must last through ages of time. Whenever and wherever anatomical studies, especially in relation to surgery are wanted, Deaver's work will be examined and be praised for its accuracy, artistic excellence and comprehensiveness of detail.

DISEASES OF THE STOMACH. A Textbook for Practitioners and Students. By MAX EINHORN, M. D., Professor of Clinical Medicine at the New York Post-Graduate Medical School and Hospital; Visiting Physician to the German Dispensary. Octavo, pp. 551. Third revised edition. Illustrated. New York; William Wood & Co. 1903. [Price, $3.50.]

The first edition of this work appeared about seven years ago. In the JOURNAL for April, 1897, p. 718, we presented a comprehensive review of the treatise, in which we referred to the work relating to diseases of the stomach which Drs. Charles

G. Stockton and Allen A. Jones, of Buffalo, were doing. At that time the literature of the subject was beginning to increase in quantity and quality, thanks not only to Stockton and Jones, but to Kinnicut, Stewart, Hemmeter, and Einhorn in this country, and to Laube, Boas, Ewald and others in Germany.

Since we wrote at the time above mentioned there has been greater progress than ever before in this branch of medicine, and much credit therefore continues to attach to the names already mentioned, as well as to some others not here recorded. This edition has been revised in all essential particulars to bring it abreast of the immediate present, and some additions to the text on this account have been required. But it is to all appearance the same book in style, size and general make-up. Einhorn is one of the foremost men in this field, and his book is in the forefront of the science, giving, as it does, all the essentials pertaining to diseases of the stomach, according to present knowledge of the subject. We have not thought it incumbent again to review the work in detail.

The International Medical Annual. A yearbook of Treatment and Practitioner's Index. Thirty-four contributors, American and Foreign. Twenty-first year. Octavo, pp. 750. New York and Chicago: E. B. Treat & Co. 1903. [Price, $3.00.]

It speaks well for this annual digest of medical science that it has now reached its twenty-first year. Few books of any kind stand the severe test of time. It was Emerson's rule to read no book that was not a year old, for, said he, at the end of that time nine-tenths of the books are dead. The original style of the volume has not been changed from the beginning. This is well, for it gives a uniformity to the appearance the group presents in the library, which is pleasing to the eye. Moreover, the general make-up of the book was so well chosen in the beginning, that very little improvement could be made.

The contents of this number is of more than usual interest. It is replete with the most modern methods of treatment in the diseases met with in everyday practice, hence will be found a valuable reference book by every physician engaged in the general practice of medicine. Besides, there are many subjects included that will interest the specialist, hence to him also it becomes a work of importance. The illustrations are many and well executed, which contributes to the usefulness of a book that is the peer of its class.

---

Clinical Treatises on the Pathology and Therapy of Disorders of Metabolism and Nutrition. By Prof. Dr. Carl von Noorden, Physician-in-Chief to the City Hospital, Frankfurt a. M. Authorised American edition, translated under the direction of Boardman Reed, M. D., Professor of Diseases of the Gastrointestinal Tract, Hygiene and Climatology, Department of Medicine, Temple College; Physician to the Samaritan Hospital, Philadelphia. Part III. Membranous Catarrh of the Intestines. By Prof. Dr. Carl von Noorden. With the collaboration of Dr. Carl Dapper. Duodecimo, pp. 64. New York: E. B. Treat & Co. 1903. [Price, 50 cents.]

This monograph deals with a disease of much importance, one, indeed, that occurs with sufficient frequency to make it of interest to the practising physician. It ought to be recognised without difficulty, the chief symptoms being the presence of mucus in the stools and pain. The treatment is excellently set forth—rest, hot applications, sedatives, oil by the mouth or in clysters, massage in the interval, and attention to diet. The book is well worth a careful study.

---

The Practical Medicine Series of Year Books. Comprising ten volumes on the year's progress in Medicine and Surgery. Issued monthly under the general editorial charge of Gustavus P. Head, M. D., Professor of Laryngology and Rhinology in the Chicago Post-Graduate Medical School. Volume IV. Gynecology. Edited by Emilius C. Dudley, A. M., M. D., Professor of Gynecology, Northwestern University Medical School. Chicago, and William Healy, A. B., M. D., Instructor in Gynecology, Northwestern University Medical School. Chicago: The Year Book Publishers. 1903. [Price, $1.25; entire series, $7.50.]

This volume, as announced by the editors, is a résumé of the literature of gynecology for the year ending February 1, 1903. The aim has been to choose only the best practical articles, such as are adapted in particular to those physicians who, besides other work, may have frequent occasion to treat the diseases peculiar to women. How well the work has been done may be determined only by a careful examination of the book. This, if we mistake not, will result in a unanimous finding, that it is altogether the best annual digest of gynecological literature that has been published up to the present time.

The sections on gynecologic examination and treatment, as a matter of course, will attract the attention of a large number of readers, and from them much may be learned that is of great practical value. We, however, would invite attentive observation to the description and illustrations concerning E. C. Dudley's operation for cystocele; also, to the plates and explanatory text relating to the technic of vaginal hysterectomy by the same author. While the book is full of interest, these two subjects in particular are of special concern, being elaborated in much detail and unusually well illustrated.

